# Circulating tumour cell enumeration does not correlate with Miller–Payne grade in a cohort of breast cancer patients undergoing neoadjuvant chemotherapy

**DOI:** 10.1007/s10549-020-05658-7

**Published:** 2020-05-06

**Authors:** Sharon A. O’Toole, Cathy Spillane, Yanmei Huang, Marie C. Fitzgerald, Brendan Ffrench, Bashir Mohamed, Mark Ward, Michael Gallagher, Tanya Kelly, Cathal O’Brien, Carmel Ruttle, Anna Bogdanska, Cara Martin, Dorinda Mullen, Elizabeth Connolly, Sarah A. McGarrigle, John Kennedy, John J. O’Leary

**Affiliations:** 1 Department of Histopathology, Trinity College Dublin and Emer Casey Molecular Pathology Research Laboratory, Coombe Women’s and Infants University Hospital, Dublin, Ireland; 2 Department of Obstetrics and Gynaecology, Trinity College, Dublin, Ireland; 3 Trinity St James’s Cancer Institute, Dublin 8, Ireland; 4 School of Forensic Medicine, Xinxiang Medical University, Xinxiang, China; 5 Cancer Molecular Diagnostics, St. James’s Hospital, Dublin 8, Ireland; 6 Department of Surgery, St James’s Hospital, Dublin 8, Ireland; 7 HOPE Directorate, St. James’s Hospital, Dublin 8, Ireland

**Keywords:** Breast cancer, Circulating tumour cells (CTCs), Neoadjuvant chemotherapy (NAC), Pathological complete response (pCR), Miller–Payne grade

## Abstract

**Purpose:**

The association between pathological complete response (pCR) in patients receiving neoadjuvant chemotherapy (NAC) for breast cancer and Circulating Tumour Cells (CTCs) is not clear. The aim of this study was to assess whether CTC enumeration could be used to predict pathological response to NAC in breast cancer as measured by the Miller–Payne grading system.

**Methods:**

Twenty-six patients were recruited, and blood samples were taken pre- and post-NAC. CTCs were isolated using the ScreenCell device and stained using a modified Giemsa stain. CTCs were enumerated by 2 pathologists and classified as single CTCs, doublets, clusters/microemboli and correlated with the pathological response as measured by the Miller–Payne grading system. *χ*^2^ or ANOVA was performed in SPSS 24.0 statistics software for associations.

**Results:**

89% of patients had invasive ductal carcinoma (IDC) and 11% invasive lobular carcinoma (ILC). At baseline 85% of patients had CTCs present, median 7 (0–161) CTCs per 3 ml of whole blood. Post-chemotherapy, 58% had an increase in CTCs. This did not correlate with the Miller–Payne grade of response. No significant association was identified between the number of CTCs and clinical characteristics; however, we did observe a correlation between pre-treatment CTC counts and body mass index, *p* < 0.05.

**Conclusions:**

Patients with a complete response to NAC still had CTCs present, suggesting enumeration is not sufficient to aid surgery stratification. Additional characterisation and larger studies are needed to further characterise CTCs isolated pre- and post-chemotherapy. Long-term follow-up of these patients will determine the significance of CTCs in NAC breast cancer patients.

**Electronic supplementary material:**

The online version of this article (10.1007/s10549-020-05658-7) contains supplementary material, which is available to authorized users.

## Introduction

Breast cancer is a major public health issue globally, representing the most common cancer in women and one in ten of all newly diagnosed cancers. It is also the main cause of female cancer death globally, with 2,088,849 cases diagnosed in 2018 and 626,679 deaths [1]. Neoadjuvant chemotherapy (NAC) is now a standard treatment for breast cancer, often shrinking the tumour and allowing a less aggressive surgical approach for the patient [2]. 7–27% of new breast cancers are treated with NAC [3]. Between 10 and 40% of patients, depending on tumour subtype, receiving NAC can have a pathologic complete response (pCR) to chemotherapy [4] as determined by the Miller–Payne grading system [5].

While mortality has decreased dramatically due to earlier diagnosis and advances in treatment, metastatic disease represents the main cause of breast cancer-related morbidity and death [6], and approximately 20% of breast cancers will experience metastatic relapse. Disease spread via circulating tumour cells in the bloodstream may explain how metastasis occurs [7, 8]. At present, enumeration of CTCs is limited in the clinical setting to predicting clinical outcome [9, 10]. However, future potential applications include their use for both determining and monitoring efficacy of personalised treatment, and predicting and detecting metastases [11, 12]. In order that the full prognostic and predictive power of CTCs is realised, there are a number of issues to examine in terms of limitations in how CTCs are defined, detected and isolated [9], as EpCAM-based detection excludes what is widely considered the most clinically relevant subsets of CTCs. The gold standard CellSearch™ for CTC identification approved by U.S. Food and Drug Administration (FDA) uses fluorescently labelled cytokeratin monoclonal antibodies, the nuclear stain DAPI and the absence of staining by CD45 (pan-leukocyte stain), resulting in the overall selection of EpCAM^+^, CK8^+^, CK18^+^, CK19^+^ and CD45^−^ cells [9]. A major disadvantage of a number of CTC detection techniques is that they are dependent on capture based on epithelial marker expression, i.e., EpCAM and cytokeratins [13]. EpCAM is not a universal CTC biomarker [14], and therefore, detection is limited if expression has been downregulated due to epithelial mesenchymal transition (EMT) [15], while circulating tumour cells of mesenchymal origin are also not captured [16]. It is well recognised that CTCs are heterogeneous, with certain subgroups of CTCs harbouring higher metastatic potential. CTC clusters/microemboli have been associated with a worse clinical outcome in breast and lung cancer [17–19]. Current technologies underestimate CTC clusters because few specialised devices exist for the detection of CTC clusters and microemboli [20, 21].

The relationship between the presence of CTCs in the circulation and the response to NAC is currently an area of interest. If CTCs had the ability to predict those patients that have a complete pathological response, this could have a major impact on breast cancer treatment. A recent meta-analysis looking at the utility of CTCs assessed using the CellSearch™ system in non-metastatic breast cancer patients receiving neoadjuvant chemotherapy did not find any correlation with CTC count and response to chemotherapy [22]. A second meta-analysis concluded that CTC count as measured by multiple devices had utility in predicting therapy response in breast cancer [23]. The isolation technologies and characterisation of CTCs are clearly recognised as a limitation in these studies.

This current study focussed on evaluation of all physical forms of CTCs, using a non-marker based approach, ScreenCell (Paris, France), pre- and post-neoadjuvant chemotherapy for breast cancer in order to ascertain the utility of CTCs to predict response to neoadjuvant chemotherapy. The ScreenCell size-selective method takes advantage of the larger size of CTCs compared with nucleated blood cells for isolation, with its circular pores of 7.5 ± 0.36 μm randomly distributed throughout the filter with a pore density of 1 × 10^5^ pores/cm^2^ [24]. It avoids the bias introduced by antibodies, and false negatives/positives associated with these methods. Response to chemotherapy was assessed using the Miller–Payne grading system [5] and radiological assessment.

## Methods

### Patient recruitment

Blood samples were obtained from 26 patients undergoing treatment for breast cancer at St. James’s Hospital, Dublin 8, Ireland, between 2015 and 2016. All patients received informed consent, and the study was approved by the St James’s and Adelaide and Meath incorporating the National Children’s Hospital research ethics committee. Patients with a preoperative indication at multidisciplinary team discussion for neoadjuvant chemotherapy followed by surgery were recruited. Patients with Stage 4 disease were excluded. A 3 ml blood sample was taken from each patient prior to initiation of chemotherapy, and following completion of chemotherapy but prior to surgery. Clinicopathological data was collected for each patient including patient age, body mass index (BMI), receptor status, tumour grade, lymphovascular space invasion (LVI), clinical and pathological stage (TNM status), pathological (Miller–Payne grade), and radiological response to chemotherapy. An Allred score was used to determine oestrogen and progesterone status. The Abbott Vysis system was used to assess HER2 status. The molecular subtype was recorded but a limitation of this is that ki-67 is not routinely done in our centre so the differentiation between luminal A and B was not always possible. The characteristics of these patients are shown in Table 1. In addition, a number of other blood parameters assessed as part of the routine clinical care including CA153 (if relevant), haemoglobin, haematocrit, white cell count and platelet count were recorded for analysis.

**Table 1:** Association between pre-chemotherapy and post-chemotherapy CTC counts and clinical characteristics

Characteristics	Patients *n* (%)	Pre-chemo CTCs patients *n* (%)		*p* value	Post-chemo CTCs patients *n* (%)		*p* value
		< 5	≥ 5		< 5	≥ 5	
Patient cohort	26 (100.0%)	11 (42%)	15 (58%)		9 (35%)	17 (65%)	
Age (median 46 years)							
< 46	13 (50.0%)	5 (19.2%)	8 (30.8%)	0.691	4 (15.4%)	9 (34.6%)	0.68
≥ 46	13 (50.0%)	6 (23.1%)	7 (26.9%)		5 (19.2%)	8 (30.8%)	
BMI							
< 25	12 (46.2%)	8 (30.8%)	4 (15.4%)	0.02*	4 (15.4%)	8 (30.8%)	0.899
≥ 25	14 (53.8%)	3 (11.5%)	11 (42.3%)		5 (11.5%)	9 (34.6%)	
Subtype							
Ductal	23 (88.5%)	9 (34.6%)	14 (53.8%)	0.364	7 (26.9%)	16 (61.5%)	0.215
Lobular	3 (11.5%)	2 (7.7%)	1 (3.8%)		2 (7.7%)	1 (3.8%)	
Receptor status							
ER+/PR+	17 (65.4%)	8 (30.8%)	9 (34.6%)	0.286	7 (26.9%)	10 (38.5%)	0.139
ER−/PR−	8 (30.8%)	2 (7.7%)	6 (23.1%)		1 (3.8%)	7 (26.9%)	
ER+/PR−	1 (3.8%)	1 (3.8%)	0 (0.0%)		1 (3.8%)	0 (0.0%)	
HER2+	4 (15.4%)	2 (7.7%)	2 (7.7%)	0.735	2 (7.7%)	2 (7.7%)	0.482
HER2−	22 (84.6%)	9 (34.6%)	13 (50.0%)		7 (26.9%)	15 (57.7%)	
TNBC	7 (26.9%)	2 (7.7%)	5 (19.2%)	0.39	1 (3.8%)	6 (23.1%)	0.186
Non-TNBC	19 (73.1%)	9 (34.6%)	10 (38.5%)		8 (30.8%)	11 (42.3%)	
Molecular subtype							
Luminal-A-like^#^	15 (57.7%)	7 (26.9%)	8 (30.8%)	0.548	6 (23.1%)	9 (34.6%)	0.339
Luminal-B (Her2+)	3 (11.5%)	2 (7.7%)	1 (3.8%)		2 (7.7%)	1 (3.8%)	
Basal like/TNBC	7 (26.9%)	2 (7.7%)	5 (19.2%)		1 (3.8%)	6 (23.1%)	
Her2 enriched	1 (3.8%)	0 (0.0%)	1 (3.8%)		0 (0.0%)	1 (3.8%)	
Grade							
Grade 1	2 (7.7%)	0 (0.0%)	2 (7.7%)	0.155	0 (0.0%)	2 (7.7%)	0.372
Grade 2	16 (61.5%)	9 (34.6%)	7 (26.9%)		7 (26.9%)	9 (34.6%)	
Grade 3	8 (30.8%)	2 (7.7%)	6 (23.1%)		2 (7.7%)	6 (23.1%)	
Clinical stage							
T1	2 (7.7%)	1 (3.8%)	1 (3.8%)	0.763	0 (0.0%)	2 (7.7%)	0.177
T2	10 (38.5%)	5 (19.2%)	5 (19.2%)		2 (7.7%)	8 (30.8%)	
T3	14 (53.8%)	5 (19.2%)	9 (34.6%)		7 (26.9%)	7 (26.9%)	
Lymph node mets pre							
Yes	20 (76.9%)	8 (30.8%)	12 (46.2%)	0.664	8 (30.8%)	12 (46.2%)	0.292
Not identified	6 (23.1%)	3 (11.5%)	3 (11.5%)		1 (3.8%)	5 (19.2%)	
Radiological response							
No response	1 (3.8%)	1 (3.8%)	0 (0.0%)	0.274	0 (0.0%)	1 (3.8%)	0.674
Partial	18 (69.2%)	6 (23.1%)	12 (46.2%)		7 (26.9%)	11 (42.3%)	
Complete	7 (26.9%)	4 (15.4%)	3 (11.5%)		2 (7.7%)	5 (19.2%)	
Pathological stage							
Tis, T0–T1	16 (61.5%)	6 (23.1%)	10 (38.5%)	0.53	4 (15.4%)	12 (46.2%)	0.192
T2–T3	10 (38.5%)	5 (19.2%)	5 (19.2%)		5 (19.2%)	5 (11.5%)	
Lymph node metastasis path							
Yes	16 (61.5%)	7 (26.9%)	9 (34.6%)	0.851	6 (23.1%)	10 (38.5%)	0.696
Not identified	10 (38.5%)	4 (15.4%)	6 (23.1%)		3 (11.5%)	7 (26.9%)	
LVI							
Yes	6 (23.1%)	4 (15.4%)	2 (7.7%)	0.285	3 (11.5%)	3 (11.5%)	0.574
NI	15 (57.7%)	6 (23.1%)	9 (34.6%)		4 (15.4%)	11 (42.3%)	
NA	5 (19.2%)	1 (3.8%)	4 (15.4%)		2 (7.7%)	3 (11.5%)	
Miller–Payne grade							
1	1 (3.8%)	1 (3.8%)	0 (0.0%)	0.113	0 (0.0%)	1 (3.8%)	0.959
2	8 (30.8%)	6 (23.1%)	2 (7.7%)		3 (11.5%)	5 (11.5%)	
3	9 (34.6%)	2 (3.8%)	7 (26.9%)		3 (11.5%)	6 (23.1%)	
4	3 (11.5%)	1 (3.8%)	2 (7.7%)		1 (3.8%)	2 (7.7%)	
5	5 (19.2%)	1 (3.8%)	4 (15.4%)		2 (7.7%)	3 (11.5%)	

Lymph Node Mets Pre (Lymph node metastasis identified on pre-treatment biopsy or imaging)

Lymph Node Metastasis Path (Lymph node metastasis identified on surgical specimen)

*BMI* Body Mass Index, *NA* non-applicable, *LVI* lymphovascular invasion, *NI* not identified, *ER* oestrogen receptor, *PR* progesterone receptor, *HER2* epidermal growth factor receptor, *TNBC* triple negative breast cancer

*P value < 0.05

^#^Luminal A and B were not differentiated in all cases as Ki-67 is not performed routinely in our centre

### Blood processing

Patient blood samples were obtained in K_2_ EDTA tubes at 4 °C. 3 ml of blood was placed in a 15-ml falcon tube, combined with 4 ml FC_2_ buffer, inverted 3 times and incubated for 8 min at room temperature (RT). Blood was filtered through the ScreenCell device as per manufacturer’s instructions.

The filter was detached from the device to enable downstream manipulation. The filter was placed on tissue paper and 50 µl PBS was drawn through twice by gentle application of pressure using tweezers on the metal O-ring. The filter was submerged in 3 ml Histoclear II and detached from the O-ring using curved-tipped callipers. A small right angle was cut on the upper left for the upper side identification. The filter was submerged 3–4 times in dH_2_O to rinse off excess Histoclear II.

### Giemsa staining and imaging

200 µl modified Giemsa was applied to the filter and incubated at RT for 10 min. 200 µl buffer pH 6.8 was applied and incubated for 2 min at RT. Buffer was removed, the washing step repeated and the filter was submerged in 3 ml fresh Histoclear II.

To prepare for imaging the filter was mounted in Histoclear II. The slides were stored in a humidified chamber and scanned using a NanoZoomer 2.0-RS (Hamamatsu Photonics KK, Japan) at 20X with 9 layer z-stacks of 2 µM per stack.

Two pathologists reviewed the filters and identified CTCs on the basis of morphology, using the following criteria: intact cell, high nuclear:cytoplasmic ratio, hyperchromatic nucleus with coarse chromatin, and the presence of macro-nucleoli. CTC clusters/microemboli are defined as ≥ 3 CTCs [19] in a spatiotemporal pattern.

### Statistical analysis

All data were analysed using SPSS 24.0 statistic software (SPSS Inc., Chicago, IL, USA). The associations between CTCs and clinical and pathological variables were evaluated with *χ*^2^ and ANOVA with *p* < 0.05 indicating significance.

## Results

### Clinicopathological data

Twenty-six patients were recruited, and blood samples were taken prior to neoadjuvant chemotherapy and post-neoadjuvant chemotherapy. Breast cancer diagnosis was made following referral by imaging (mammography, ultrasound, magnetic resonance imaging (MRI)) and biopsy. Disease was staged and the presence of metastatic disease assessed via Computerised Tomography (CT)/Thorax, Abdomen, Pelvis (TAP) and bone scan. Clinicopathological details are presented in Tables 1 and S1. The median age was 46 (29–69) years. Median BMI was 27 (18–38), with over 50% of the cohort in the overweight/obese category. 89% (23) of the patients were diagnosed with invasive ductal carcinoma (IDC) and 11% of patients (3) were diagnosed with invasive lobular carcinoma (ILC). Patients received neoadjuvant chemotherapy following discussion at a multidisciplinary team meeting. The majority of patients had locally advanced disease with no distant metastasis, while others had a triple positive or triple negative diagnosis with no lymph node metastasis diagnosis prior to treatment. Patients were treated with the ACT chemotherapy regimen, which consists of doxorubicin (Adriamycin) and cyclophosphamide, followed by treatment with paclitaxel (taxane). Patients with human epidermal growth factor receptor 2 (HER2^+^) tumours also received Herceptin®. One patient developed neuropathy and did not complete paclitaxel treatment. Response to neoadjuvant chemotherapy was assessed prior to surgery using ultrasound, mammography or MRI. 65% expressed oestrogen receptor (ER) and progesterone receptor (PR) as displayed in Tables 1 and S1. Four (15%) of the cohort expressed HER2 which was confirmed by fluorescence in situ hybridisation (FISH). The predominant molecular subtype in our cohort was ER^+^, PR^+^ and HER2^−^ with 58% (15) staining for this subtype. 8% were triple positive and 27% triple negative. Pathological stage was recorded post-surgery. Patient age, BMI, tumour subtype, receptor status, molecular subtype, tumour grade, clinical stage, pathological stage, LVI, radiological response and pathological response (Miller–Payne grade) to chemotherapy are displayed in Table 1.

### CTC identification and enumeration

**Fig. 1:**
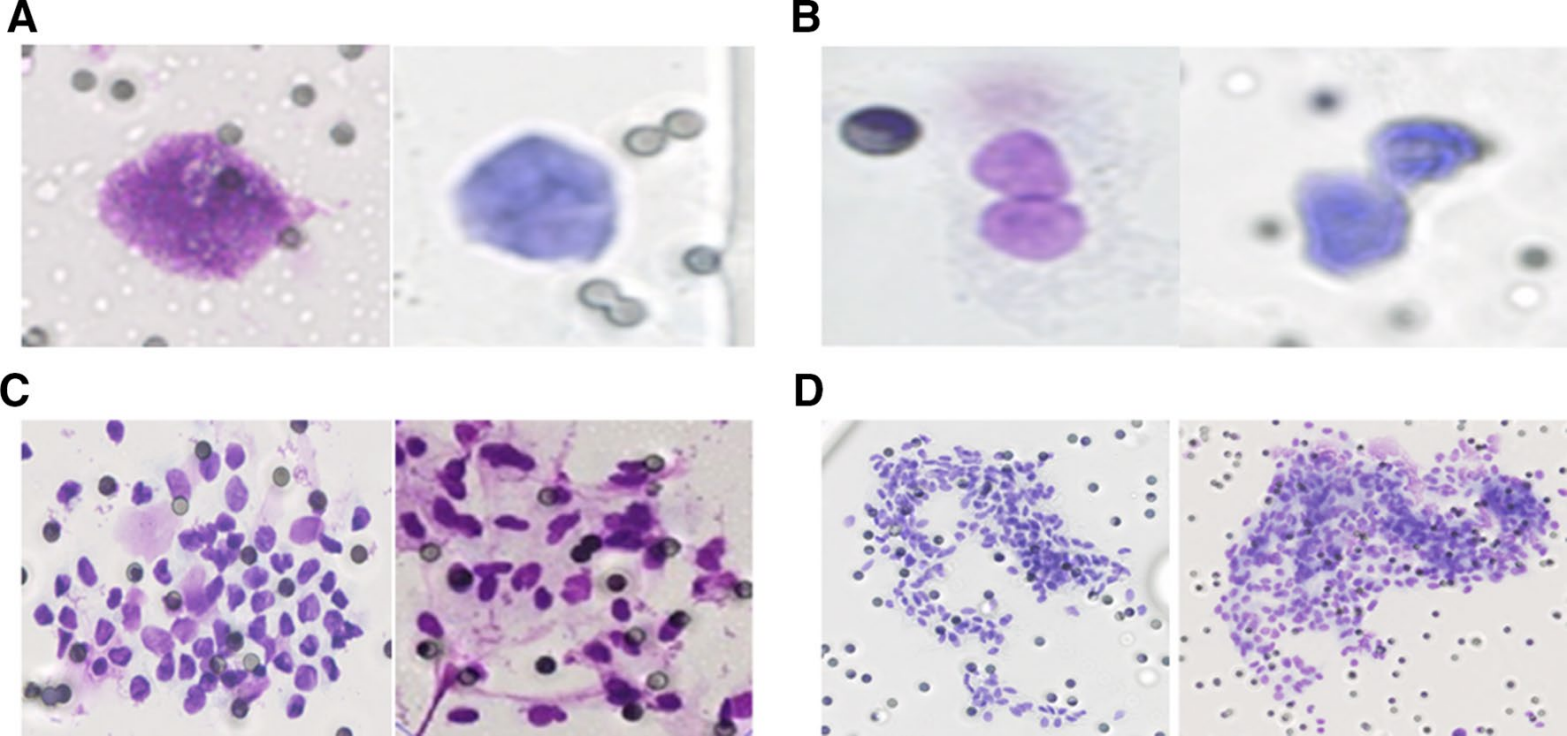
Multiple CTC physical forms were isolated from breast cancer patients. CTCs were enumerated by 2 pathologists and classified as **a** single cells, **b** doublets, **c**, **d** clusters/microemboli

Modified Giemsa staining was used for the identification of CTCs. CTC heterogeneity was observed with CTCs being identified as single cells (Fig. 1A), doublets (Fig. 1B) and clusters/microemboli (Fig. 1C, D). CTCs were enumerated in the pre- and post-chemotherapy sample and classified according to the CellSearch™ cut-off of < 5 or ≥ 5 CTCs; correlations with clinical parameters are shown in Table 1. Detailed CTC counts are displayed in Supplementary Table 1. As only 3 ml of blood was used with the ScreenCell device, correlations were also assessed with the equivalent cut-off of 2 CTCs to correct for the volume used with the CellSearch™ device and a positive or negative count (data not presented) but no significant associations were seen.

### Correlation of CTCs with clinicopathological data

Data for CTC counts are presented in Table 1 and individual counts are detailed in Supplementary Table 1. At baseline, 4 patients were CTC-negative. 7 patients had 1–4 CTCs and 15 patients had ≥ 5. Median baseline CTC count was 7 (0–161). Post -chemotherapy, 1 patient was CTC-negative, 8 patients had 1–4 CTCs and 17 patients had ≥ 5 CTCs with a median count of 9.5 (0–300).

No significant association was identified between the number of CTCs (categorised < or ≥ 5 CTCs as per the CellSearch™ studies) in the pre-chemotherapy or post-chemotherapy blood sample and clinical characteristics (Table 1). A significant correlation was observed between BMI and pre-treatment CTC count, *p* < 0.05. CTC counts were also categorised using 2 CTCs as the cut-off (correcting for the blood volume used) and as positive/negative but no correlation was observed. No correlation was found between CTC counts and haemoglobin level, haematocrit, white cell count, CA153 if available and platelet count (data not shown).

The change in total CTCs between the pre- and post-neoadjuvant chemotherapy samples was very variable between patients (Supplementary Fig. 1), with some having a decrease of 61 CTCs while others had an increase of 270 CTCs. Overall, 13 (50%) patients had an increase in total CTCs, 10 a decrease and 2 had no change.

65% of patients had a good response to neoadjuvant chemotherapy with a Miller–Payne grade of 3 or more. Five patients (19%) had a Miller–Payne grade of 5, or a complete pathological response to chemotherapy. This did not correlate with CTC counts, either pre- or post-chemotherapy or with the change in CTC numbers.

### Correlation of CTC clusters with clinicopathological data

No correlation was seen between the number of CTC clusters (also assessed as positive or negative) and clinicopathological details. However, if we categorise clusters as < or ≥ 2 we do observe a correlation with pre-treatment counts and BMI, *p* < 0.05. The number of cells in each cluster was also assessed as displayed in Supplementary Table 1 but no correlation was observed with clinical parameters including haemoglobin level, haematocrit, white cell count, CA153 if available and platelet count.

At baseline, 13 patients were negative for clusters, 5 patients had 1–4 CTC clusters and 8 patients had ≥ 5 CTC clusters. The median baseline number of clusters was 0.5 (0–40). The change in CTC clusters was again very variable between patients (Supplementary Fig. 2). Post-chemotherapy, 10 patients were negative for CTC clusters, 9 patients had < 5 clusters and 7 patients had > 5 clusters. The median post-chemotherapy CTC cluster value was 1.5 (0–26). Overall, 11 (42%) patients experienced an increase in CTC clusters, 10 had a decrease in CTC clusters and 5 remained unchanged.

### Correlation of CTCs with clinical outcome

We were unable to assess the prognostic potential of the CTC counts in our study to date due to low recurrence rate. Observational data is outlined in Supplementary Table 1. Following a 3-year follow-up, 21 patients are currently alive with no evidence of disease (ANED), 3 patients have had a recurrence (AWD) and 2 patients have died of disease (DOD). Of the 2 patients that died of disease, both had an increase in CTCs following chemotherapy, 1 had no CTCs at baseline. Of the 3 patients that had a recurrence, 1 had a decrease in CTCs post-chemo, 1 had no change in CTCs between the pre- and post-chemo sample and the third had an increase in CTCs (in particular in clusters). At this stage it would seem that the presence of CTCs post-chemotherapy will have some prognostic potential, but it is not possible to reliably say this at this point. It is our intention to review this data for a 5-year follow-up and beyond if necessary. While the data are provisional and the sample cohort is small, it does warrant a basis for future larger studies to address this question.

## Discussion

This study did not show any benefit for CTC counts prior to treatment or prior to surgery in assessing pathological response to neoadjuvant chemotherapy in breast cancer patients. It is likely that more in depth analysis of CTCs and larger studies will unlock their true potential in the clinic but currently many limitations exist in terms of their isolation and characterisation.

CTCs were detected in 85% of patients in this study prior to treatment, which is higher than some of the quoted studies in the literature which vary from 31 to 61% [22, 23]. However, many of the published studies used the CellSearch™ system, which we know underestimates CTC numbers due to its reliance on the presence of EpCAM. In addition, some meta-analyses published [22, 23] have included early breast cancer patients whereas in this study we focussed on those with locally advanced disease (including some triple positive/negative with N0 disease *n* = 6) who were undergoing NAC followed by surgery, a cohort we would expect to be higher CTC traffickers. The GeparQuattro trial did focus on a neoadjuvant cohort using the CellSearch™ system and found prognostic ability in pre-treatment CTC counts for HER2 positive and triple negative patients using a cut-off of 2 CTCs but similar to our findings this prognostic ability was independent of the primary tumour response [25]. The patient population selected and the ScreenCell isolation device used may explain the higher percentage of patients having CTCs at the outset.

Significant heterogeneity was observed in the CTC phenotypes isolated, single cells, doublets and clusters. At baseline, clusters were isolated in 50% of our cohort. This is slightly higher than recently reported by Vetter et al., who found 35% of their cohort had clusters present prior to treatment [26], using the Parsortix microfluidic device. Studies using the CellSearch™ system report cluster rates around 20% [27]. Current technologies significantly under-call the number of clusters because few specialised devices exist for their detection. The definition of clusters also varies in the literature from > 1 CTC to > 2 or 3 cells [19]. In this study, we define clusters as ≥ 3 CTCs but we have also conducted analyses using the other cut-offs of > 1/2 cells but no significance was seen. Clusters are more aggressive than single CTCs [18, 19] and a lot of effort is now focussed on their isolation and characterisation [20, 21]. We have previously reported the importance of platelets in cancer metastasis, which are a major component of these clusters and microemboli [28] and may explain the increased aggressiveness observed. In addition, we have shown that the platelet cloak can inhibit immune surveillance by NK cells enabling the CTCs to establish metastasis [29].

No association was seen between CTC counts and clinicopathological details apart from BMI and pre-treatment CTC counts. BMI has been shown to mediate the prognostic significance of CTCs in inflammatory breast cancer [30]. In a recent publication, patient-derived xenograft (PDX) model, tumours grown in the presence of obesity-altered adipose stem cells in SCID/beige mice had increased circulating HLA1^+^ human cells as well as increased numbers of CD44^+^CD24^−^ cancer stem cells in the peripheral blood, the authors concluded leptin produced by obesity-altered adipose stem cells promotes metastasis. Others have found a negative association with BMI and CTC counts using the CellSearch device [31, 32]. Further work is needed to assess this correlation between CTCs and BMI. Most studies including two meta-analyses [22, 23], did not observe any correlation with clinicopathological details but they did find CTC counts to have prognostic ability. The prognostic potential of CTC counts in our study has not yet been statistically assessed due to the low number of patients, presenting with recurrences but it is our intention to carry out a 5-year follow-up on these patients. It does, however, seem from the observational data that the presence of CTCs post-chemotherapy may have some prognostic potential.

Some studies have assessed correlation of CTC counts with complete pathological response. The method of measuring the pCR is not detailed in all studies. In this study, the CTC count before and after chemotherapy was correlated with radiological findings and also to the pathological score of Miller–Payne grading but no correlation was seen, 19% of the patients in our cohort had a pCR and all still had CTCs following neoadjuvant chemotherapy. Other studies report a decline in CTC counts post-neoadjuvant treatment [33] while some report an increase [34]. While CTCs were present post-treatment, we do not know the metastatic potential of these CTCs and this is needed to unlock the true potential of CTCs in the clinic. In our study, we observed an increase of 58% in CTCs and 42% in CTC clusters in the patients post-chemotherapy which is similar to the observations in a prostate cancer study where 42% of patients had an increase in CTCs post-treatment [35]. There may be many reasons for the increase in CTC counts/clusters observed post-treatment. This may be explained by the rate at which the various tumour subtypes shed tumour cells into the circulation. The chemotherapy may have caused the vessels to become leaky and shed more cells into the vasculature. It may be that the chemotherapy has selected for a clonal population of stem like cells that have survived the chemotherapy. Other mechanisms that may explain this increased number include autophagy, aniokis, expansion of pro-metastatic variants or a potential drive towards dormancy, which would be of interest for future studies. Clearly, further work is needed on assessing the viability and molecular characterisation of the CTCs that remain post-treatment. We hypothesise that CTC clusters are more likely to be viable than single CTCs. While these reasons may explain the findings in the cohort of patients who still have residual disease, it does not explain the situation for those who have had a complete pathological response. It suggests a differential response between the primary tumour and the CTCs and is similar to the findings in the Geparquattro trial [18]. Follow-up of these patients is needed to determine the long-term significance of these CTCs. One study has reported the detection of CTCs 8–22 years out from treatment, despite no clinical evidence of disease [36]. It is not known if this represents tumour dormancy and persistence of disseminated disease in the bone marrow as suggested by some investigators [37, 38] or whether a proportion of these patients will go on to develop metastatic disease. This stresses the need for molecular characterisation and long-term follow-up of patients.

Many groups are now focussing on characterising and dissecting the metastatic potential of CTCs. Our group has established a CTC-5 program which allows us to merge a morphology image of the isolated CTCs with an immunofluorescent profile for the same cells which will give us a better insight into their biology (manuscript in prep). A focus on single cell genomics and cluster dissection may reveal mechanisms of how the cells in a cluster co-operate and metastasise, enabling CTCs to have a more proactive role in the clinic. Unlocking the full potential of the liquid biopsy to monitor treatment response in breast cancer will allow us ultimately to deliver a more personalised medicine approach for cancer patients, improving therapeutic outcomes.

## Conclusions

The finding that CTCs still exist post-neoadjuvant chemotherapy in patients who have a complete pathological response is a significant finding in the setting of what this study was trying to achieve, demonstrating that CTC enumeration may not be suitable to determine if patients can avoid surgery. Larger studies will be needed to further evaluate this finding. Additional characterisation of CTCs and CTC clusters is needed to assess the true potential of CTCs in this cohort of patients. Long-term follow-up of patients is needed to assess the significance of CTC counts.

## Electronic supplementary material

Below is the link to the electronic supplementary material.

Supplementary Figure 1: Change in CTC count between pre-chemotherapy and post-chemotherapy blood samples. Legend: Pre-chemotherapy total CTC counts are displayed as square and post-chemotherapy counts as circles (PNG 296 kb)

Supplementary Figure 2: Change in CTC cluster count between pre-chemotherapy and post-chemotherapy blood samples. Legend: Pre-chemotherapy cluster numbers are displayed as square and post-chemotherapy cluster numbers as circles (PNG 276 kb)

Supplementary Table S1 Clinicopathological characteristics and CTC counts (XLSX 18 kb)

## Abbreviations

ANED: Alive with no evidence of disease
AWD: Alive with disease
BL: Baseline
BMI: Body mass index
CK: Cytokeratin
CT: Computerised tomography
CTCs: Circulating tumour cells
DOD: Died of disease
EMT: Epithelial-mesenchymal transition
EpCAM: Epithelial cell adhesion molecule
ER: Oestrogen receptor
FISH: Fluorescence in situ hybridisation
HER2: Human epidermal growth factor receptor 2
HR: Hazard ratio
IDC: Invasive ductal carcinoma
ILC: Invasive lobular carcinoma
MRI: Magnetic resource imaging
NAC: Neoadjuvant chemotherapy
OS: Overall survival
pCR: Pathological complete response
PR: Progesterone receptor
RT: Room temperature
TNBC: Triple-negative breast cancer

## References

[1] Bray F, Ferlay J, Soerjomataram I, Siegel RL, Torre LA, Jemal A (2018). Global cancer statistics 2018: GLOBOCAN estimates of incidence and mortality worldwide for 36 cancers in 185 countries. CA Cancer J Clin.

[2] Kaufmann M, von Minckwitz G, Bear HD, Buzdar A, McGale P, Bonnefoi H, Colleoni M, Denkert C, Eiermann W, Jackesz R, Makris A, Miller W, Pierga JY, Semiglazov V, Schneeweiss A, Souchon R, Stearns V, Untch M, Loibl S (2007). Recommendations from an international expert panel on the use of neoadjuvant (primary) systemic treatment of operable breast cancer: new perspectives 2006. Ann Oncol.

[3] Vaidya JS, Massarut S, Vaidya HJ, Alexander EC, Richards T, Caris JA, Sirohi B, Tobias JS (2018). Rethinking neoadjuvant chemotherapy for breast cancer. BMJ.

[4] Houssami N, Macaskill P, von Minckwitz G, Marinovich ML, Mamounas E (2012). Meta-analysis of the association of breast cancer subtype and pathologic complete response to neoadjuvant chemotherapy. Eur J Cancer.

[5] Ogston KN, Miller ID, Payne S, Hutcheon AW, Sarkar TK, Smith I, Schofield A, Heys SD (2003). A new histological grading system to assess response of breast cancers to primary chemotherapy: prognostic significance and survival. Breast.

[6] Jemal A, Bray F, Center MM, Ferlay J, Ward E, Forman D (2011). Global cancer statistics. CA Cancer J Clin.

[7] Giuliano M, Giordano A, Jackson S, De Giorgi U, Mego M, Cohen EN, Gao H, Anfossi S, Handy BC, Ueno NT, Alvarez RH, De Placido S, Valero V, Hortobagyi GN, Reuben JM, Cristofanilli M (2014). Circulating tumor cells as early predictors of metastatic spread in breast cancer patients with limited metastatic dissemination. Breast Cancer Res.

[8] Nguyen DX, Bos PD, Massague J (2009). Metastasis: from dissemination to organ-specific colonization. Nat Rev Cancer.

[9] Millner LM, Linder MW, Valdes R (2013). Circulating tumor cells: a review of present methods and the need to identify heterogeneous phenotypes. Ann Clin Lab Sci.

[10] Heitzer E, Haque IS, Roberts CES, Speicher MR (2018). Current and future perspectives of liquid biopsies in genomics-driven oncology. Nat Rev Genet.

[11] Selli C, Dixon JM, Sims AH (2016). Accurate prediction of response to endocrine therapy in breast cancer patients: current and future biomarkers. Breast Cancer Res.

[12] Hwang WL, Pleskow HM, Miyamoto DT (2018). Molecular analysis of circulating tumors cells: Biomarkers beyond enumeration. Adv Drug Deliv Rev.

[13] Krebs MG, Hou JM, Ward TH, Blackhall FH, Dive C (2010). Circulating tumour cells: their utility in cancer management and predicting outcomes. Ther Adv Med Oncol.

[14] Gupta V, Jafferji I, Garza M, Melnikova VO, Hasegawa DK, Pethig R, Davis DW (2012). ApoStream(), a new dielectrophoretic device for antibody independent isolation and recovery of viable cancer cells from blood. Biomicrofluidics.

[15] Gorges TM, Tinhofer I, Drosch M, Rose L, Zollner TM, Krahn T, von Ahsen O (2012). Circulating tumour cells escape from EpCAM-based detection due to epithelial-to-mesenchymal transition. BMC Cancer.

[16] Gabriel MT, Calleja LR, Chalopin A, Ory B, Heymann D (2016). Circulating tumor cells: a review of non-EpCAM-based approaches for cell enrichment and isolation. Clin Chem.

[17] Mu Z, Wang C, Ye Z, Austin L, Civan J, Hyslop T, Palazzo JP, Jaslow R, Li B, Myers RE, Jiang J, Xing J, Yang H, Cristofanilli M (2015). Prospective assessment of the prognostic value of circulating tumor cells and their clusters in patients with advanced-stage breast cancer. Breast Cancer Res Treat.

[18] Aceto N, Bardia A, Miyamoto DT, Donaldson MC, Wittner BS, Spencer JA, Yu M, Pely A, Engstrom A, Zhu H, Brannigan BW, Kapur R, Stott SL, Shioda T, Ramaswamy S, Ting DT, Lin CP, Toner M, Haber DA, Maheswaran S (2014). Circulating tumor cell clusters are oligoclonal precursors of breast cancer metastasis. Cell.

[19] Hou JM, Krebs MG, Lancashire L, Sloane R, Backen A, Swain RK, Priest LJ, Greystoke A, Zhou C, Morris K, Ward T, Blackhall FH, Dive C (2012). Clinical significance and molecular characteristics of circulating tumor cells and circulating tumor microemboli in patients with small-cell lung cancer. J Clin Oncol.

[20] Jiang X, Wong KHK, Khankhel AH, Zeinali M, Reategui E, Phillips MJ, Luo X, Aceto N, Fachin F, Hoang AN, Kim W, Jensen AE, Sequist LV, Maheswaran S, Haber DA, Stott SL, Toner M (2017). Microfluidic isolation of platelet-covered circulating tumor cells. Lab Chip.

[21] Sarioglu AF, Aceto N, Kojic N, Donaldson MC, Zeinali M, Hamza B, Engstrom A, Zhu H, Sundaresan TK, Miyamoto DT, Luo X, Bardia A, Wittner BS, Ramaswamy S, Shioda T, Ting DT, Stott SL, Kapur R, Maheswaran S, Haber DA, Toner M (2015). A microfluidic device for label-free, physical capture of circulating tumor cell clusters. Nat Methods.

[22] Bidard FC, Michiels S, Riethdorf S, Mueller V, Esserman LJ, Lucci A, Naume B, Horiguchi J, Gisbert-Criado R, Sleijfer S, Toi M, Garcia-Saenz JA, Hartkopf A, Generali D, Rothe F, Smerage J, Muinelo-Romay L, Stebbing J, Viens P, Magbanua MJM, Hall CS, Engebraaten O, Takata D, Vidal-Martinez J, Onstenk W, Fujisawa N, Diaz-Rubio E, Taran FA, Cappelletti MR, Ignatiadis M, Proudhon C, Wolf DM, Bauldry JB, Borgen E, Nagaoka R, Caranana V, Kraan J, Maestro M, Brucker SY, Weber K, Reyal F, Amara D, Karhade MG, Mathiesen RR, Tokiniwa H, Llombart-Cussac A, Meddis A, Blanche P, d'Hollander K, Cottu P, Park JW, Loibl S, Latouche A, Pierga JY, Pantel K (2018). Circulating tumor cells in breast cancer patients treated by neoadjuvant chemotherapy: a meta-analysis. J Natl Cancer Inst.

[23] Yan WT, Cui X, Chen Q, Li YF, Cui YH, Wang Y, Jiang J (2017). Circulating tumor cell status monitors the treatment responses in breast cancer patients: a meta-analysis. Sci Rep.

[24] Desitter I, Guerrouahen BS, Benali-Furet N, Wechsler J, Janne PA, Kuang Y, Yanagita M, Wang L, Berkowitz JA, Distel RJ, Cayre YE (2011). A new device for rapid isolation by size and characterization of rare circulating tumor cells. Anticancer Res.

[25] Riethdorf S, Muller V, Loibl S, Nekljudova V, Weber K, Huober J, Fehm T, Schrader I, Hilfrich J, Holms F, Tesch H, Schem C, von Minckwitz G, Untch M, Pantel K (2017). Prognostic impact of circulating tumor cells for breast cancer patients treated in the neoadjuvant "Geparquattro" trial. Clin Cancer Res.

[26] Vetter M, Landin J, Szczerba BM, Castro-Giner F, Gkountela S, Donato C, Krol I, Scherrer R, Balmelli C, Malinovska A, Zippelius A, Kurzeder C, Heinzelmann-Schwarz V, Weber WP, Rochlitz C, Aceto N (2018). Denosumab treatment is associated with the absence of circulating tumor cells in patients with breast cancer. Breast Cancer Res.

[27] Larsson AM, Jansson S, Bendahl PO, Levin Tykjaer Jorgensen C, Loman N, Graffman C, Lundgren L, Aaltonen K, Ryden L (2018). Longitudinal enumeration and cluster evaluation of circulating tumor cells improve prognostication for patients with newly diagnosed metastatic breast cancer in a prospective observational trial. Breast Cancer Res.

[28] Egan K, Crowley D, Smyth P, O'Toole S, Spillane C, Martin C, Gallagher M, Canney A, Norris L, Conlon N, McEvoy L, Ffrench B, Stordal B, Keegan H, Finn S, McEneaney V, Laios A, Ducree J, Dunne E, Smith L, Berndt M, Sheils O, Kenny D, O'Leary J (2011). Platelet adhesion and degranulation induce pro-survival and pro-angiogenic signalling in ovarian cancer cells. PLoS ONE.

[29] Cluxton CD, Spillane C, O'Toole SA, Sheils O, Gardiner CM, O'Leary JJ (2019). Suppression of Natural Killer cell NKG2D and CD226 anti-tumour cascades by platelet cloaked cancer cells: implications for the metastatic cascade. PLoS ONE.

[30] Fayanju OM, Hall CS, Bauldry JB, Karhade M, Valad LM, Kuerer HM, DeSnyder SM, Barcenas CH, Lucci A (2017). Body mass index mediates the prognostic significance of circulating tumor cells in inflammatory breast cancer. Am J Surg.

[31] Sabol RA, Bowles AC, Cote A, Wise R, O'Donnell B, Matossian MD, Hossain FM, Burks HE, Del Valle L, Miele L, Collins-Burow BM, Burow ME, Bunnell BA (2019). Leptin produced by obesity-altered adipose stem cells promotes metastasis but not tumorigenesis of triple-negative breast cancer in orthotopic xenograft and patient-derived xenograft models. Breast Cancer Res.

[32] Lohmann AE, Dowling RJO, Ennis M, Amir E, Elser C, Brezden-Masley C, Vandenberg T, Lee E, Fazaee K, Stambolic V, Goodwin PJ, Chang MC (2018). Association of metabolic, inflammatory, and tumor markers with circulating tumor cells in metastatic breast cancer. JNCI Cancer Spectrosc.

[33] Martin M, Custodio S, de Las Casas ML, Garcia-Saenz JA, de la Torre JC, Bellon-Cano JM, Lopez-Tarruella S, Vidaurreta-Lazaro M, de la Orden V, Jerez Y, Marquez-Rodas I, Casado A, Sastre J, Diaz-Rubio E (2013). Circulating tumor cells following first chemotherapy cycle: an early and strong predictor of outcome in patients with metastatic breast cancer. Oncologist.

[34] Rack B, Schindlbeck C, Juckstock J, Andergassen U, Hepp P, Zwingers T, Friedl TW, Lorenz R, Tesch H, Fasching PA, Fehm T, Schneeweiss A, Lichtenegger W, Beckmann MW, Friese K, Pantel K, Janni W, Group SS (2014). Circulating tumor cells predict survival in early average-to-high risk breast cancer patients. J Natl Cancer Inst.

[35] Lorente D, Olmos D, Mateo J, Dolling D, Bianchini D, Seed G, Flohr P, Crespo M, Figueiredo I, Miranda S, Scher HI, Terstappen L, de Bono JS (2018). Circulating tumour cell increase as a biomarker of disease progression in metastatic castration-resistant prostate cancer patients with low baseline CTC counts. Ann Oncol.

[36] Meng S, Tripathy D, Frenkel EP, Shete S, Naftalis EZ, Huth JF, Beitsch PD, Leitch M, Hoover S, Euhus D, Haley B, Morrison L, Fleming TP, Herlyn D, Terstappen LW, Fehm T, Tucker TF, Lane N, Wang J, Uhr JW (2004). Circulating tumor cells in patients with breast cancer dormancy. Clin Cancer Res.

[37] Braun S, Kentenich C, Janni W, Hepp F, de Waal J, Willgeroth F, Sommer H, Pantel K (2000). Lack of effect of adjuvant chemotherapy on the elimination of single dormant tumor cells in bone marrow of high-risk breast cancer patients. J Clin Oncol.

[38] Muller V, Stahmann N, Riethdorf S, Rau T, Zabel T, Goetz A, Janicke F, Pantel K (2005). Circulating tumor cells in breast cancer: correlation to bone marrow micrometastases, heterogeneous response to systemic therapy and low proliferative activity. Clin Cancer Res.

