# Supplementary figures and images for: Circulating tumour cell enumeration does not correlate with Miller–Payne grade in a cohort of breast cancer patients undergoing neoadjuvant chemotherapy
Source: Breast Cancer Res Treat. 2020 May 6;181(3):571–80. doi: 10.1007/s10549-020-05658-7 (PMC7220879; doi:10.1007/s10549-020-05658-7)

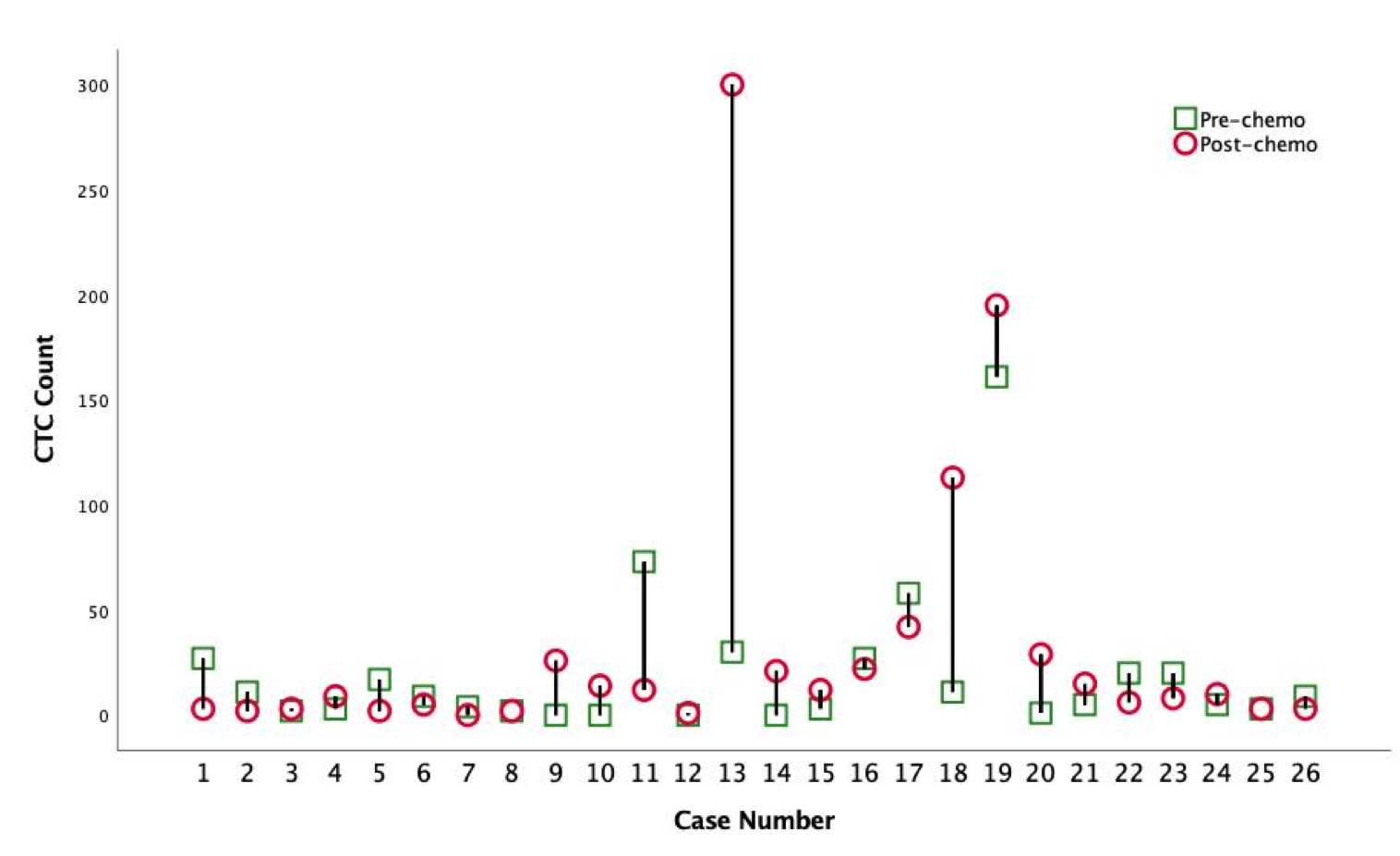

Supplement: Supplementary file 1 — Supplementary Figure 1: Change in CTC count between pre-chemotherapy and post-chemotherapy blood samples. Legend: Pre-chemotherapy total CTC counts are displayed as square and post-chemotherapy counts as circles (PNG 296 kb) [file 10549_2020_5658_MOESM1_ESM.png]

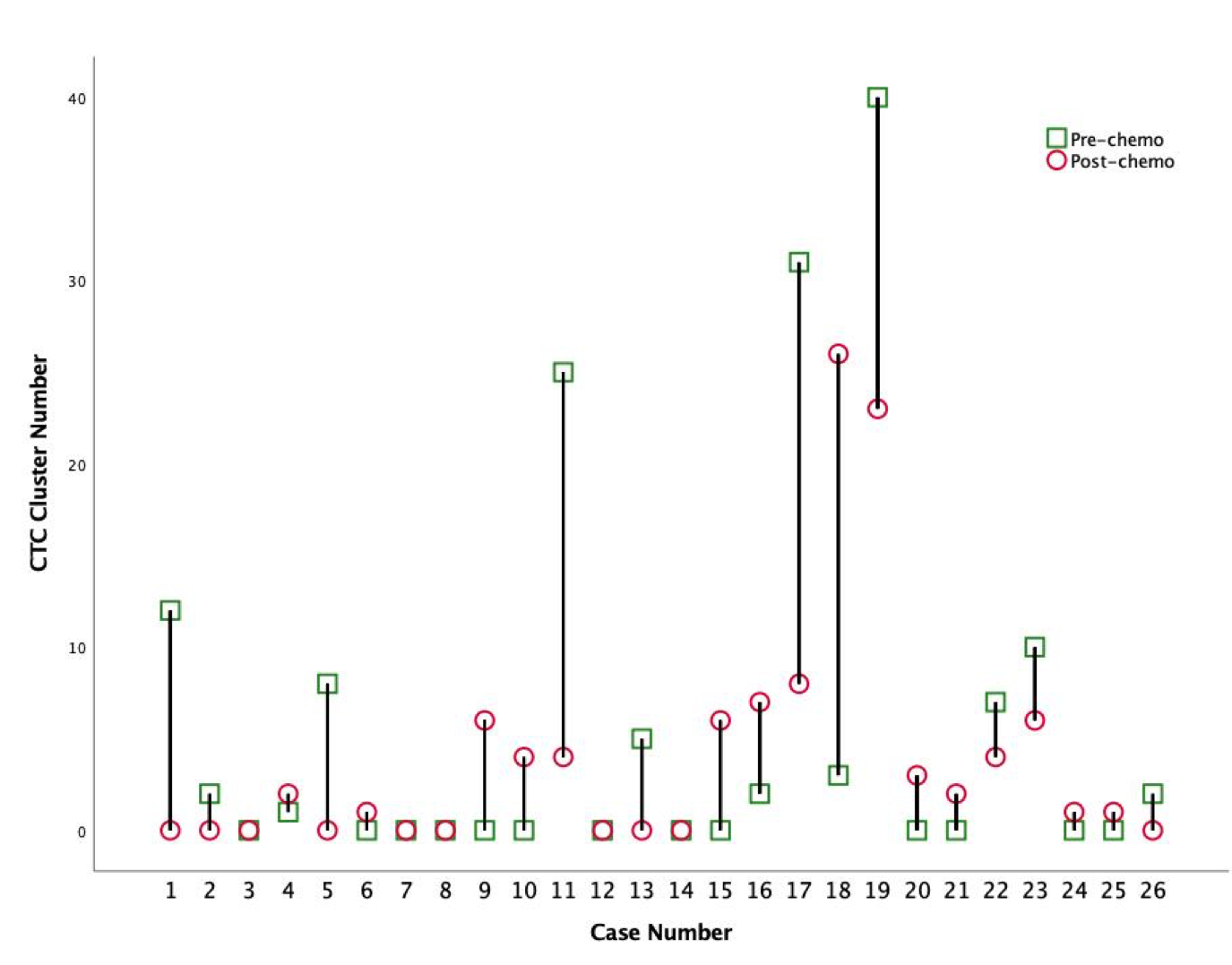

Supplement: Supplementary file 2 — Supplementary Figure 2: Change in CTC cluster count between pre-chemotherapy and post-chemotherapy blood samples. Legend: Pre-chemotherapy cluster numbers are displayed as square and post-chemotherapy cluster numbers as circles (PNG 276 kb) [file 10549_2020_5658_MOESM2_ESM.png]
